# Physicochemical and Functional Properties of Chemically Pretreated *Ndou *Sweet Potato Flour

**DOI:** 10.1155/2019/4158213

**Published:** 2019-11-03

**Authors:** Khuthadzo Ngoma, Mpho E. Mashau, Henry Silungwe

**Affiliations:** Department of Food Science and Technology, School of Agriculture, University of Venda, Private Bag X5050, Thohoyandou 0950, South Africa

## Abstract

Sweet potato (*Ipomoea batatas Lam*) is a nutritious crop abundant in calories and bioactive compounds such as beta-carotene, ascorbic acid, polyphenols, and dietary fibre. This study investigated the effect of pretreatments on the physicochemical and functional properties of *Ndou *sweet potato (NSP) flour. Flour samples were prepared by randomly assigning NSP slices to two treatments (citric acid and sodium metabisulphite) at 5, 10, and 15 g/L concentration for 10 min. Distilled water was used as control. The moisture content (7.70%) of NSP flour treated with citric acid was significantly (*p* < 0.05) higher than that of the flour treated with sodium metabisulphite (5.54%) as the concentration level increased. The treatments did not significantly (*p* < 0.05) affect the protein and fat contents of the NSP flour and protein increased from 2.54 to 2.82%, while fat decreased from 0.69 to 0.61%. Sodium metabisulphite treated samples had a higher *L*^∗^ value, ash, and pH level than citric acid treated samples. However, pH was slightly decreased by both treatments from 6.05 to 5.09. Citric acid treated samples had higher *a*^∗^ and *b*^∗^ values than sodium metabisulphite treated samples. In terms of the functional properties of NSP flour, the treatments significantly (*p* < 0.05) affected the water absorption capacity, viscosity, swelling power, solubility index, and thermal properties although the bulk density and least gelation concentration were not significantly (*p* > 0.05) affected. Sodium metabisulphite was very effective in improving physicochemical and functional properties of NSP flour as compared to citric acid. The findings of this study show the possibilities of using NSP flour in food systems as gelling agent, fat replacer, and thickeners.

## 1. Introduction

Sweet potato (*Ipomoea batatas Lam*) has been identified as a food security crop because it contains reasonable amounts of bioactive compounds such as b-carotene, ascorbic acid, polyphenols, dietary fibre as well as vitamins, minerals, and proteins [1, 2]. The crop is still underutilised although it is rich in starch and carbohydrates and its flour can be used in different industrial applications [3, 4]. The most commonly obtainable sweet potato varieties have purple, yellow, and white root tubers because of the distinct contents of phenolic compounds and pigments in their root tubers [5–7]. Processing of sweet potato roots into food products such as flour or chips can be used as one of the ways to address the challenges faced with storing and transporting the raw sweet potato roots in developing countries. *Ndou* is one of the sweet potato cultivars grown in Limpopo province, South Africa. *Ndou* sweet potato (NSP) cultivar is high-yielding and is mostly bred by the Agricultural Research Council in South Africa. It has a dark cream skin colour and flesh colour as well as a round and long elliptic storage root shape.

There are several drawbacks that have been identified during the preparation of sweet potato products and one of these is discoloration which affects the quality of products [8]. Various methods such as blanching with sodium tripolyphosphate (Na_5_P_3_O_10_) and sodium metabisulphite (Na_2_S_2_O_5_) have been used to prevent or eliminate discolouration of sweet potato flour [9, 10]. Processing sweet potato into flour extends its storage life and value. The flour has different applications in the food industry such as serving as a thickening agent in gravy, soup, bakery products, and fabricated snacks [10]. However, high quality sweet potato flour will be needed if the flour is going to be incorporated into other food products. However, there is lack of knowledge and understanding of physicochemical and functional properties of NSP and the effect that the different processing methods have on these properties and functionality of the different components. The closure of this knowledge gap may broaden the applications of NSP flour within the food industry. Sodium metabisulphite and citric acid have been used to pretreat sweet potato products, but their effects on NSP flour have not been revealed. Therefore, the aim of this work was to determine the effect of application of chemical agents: sodium metabisulphite and citric acid as pretreatments to fresh cut NSP on the physicochemical and functional properties of its flour. Processing sweet potato into flour, which is an intermediate product, is one of the ways of minimizing post-harvest losses and increasing the utilization of sweet potatoes [11].

## 2. Materials and Methods

### 2.1. Materials

Fresh NSP (*Ipomoea batatas Lam*) tubers were purchased from ARC in Pretoria, Gauteng province, South Africa. Chemicals used in this study were commercially purchased from Sigma-Aldrich, Johannesburg, South Africa.

### 2.2. Experimental Design

The experiment was set up as a completely randomised design and the pretreatments of NSP flour with sodium metabisulphite and citric acid at different concentrations were considered. The factor levels were: distilled water 5, 10, 15 g/L of sodium metabisulphite for 10 min (experiment 1) and 5, 10, 15 g/L of citric acid for 10 min (experiment 2). Each experiment was done in triplicate.

### 2.3. Flour Production

**Figure 1:**
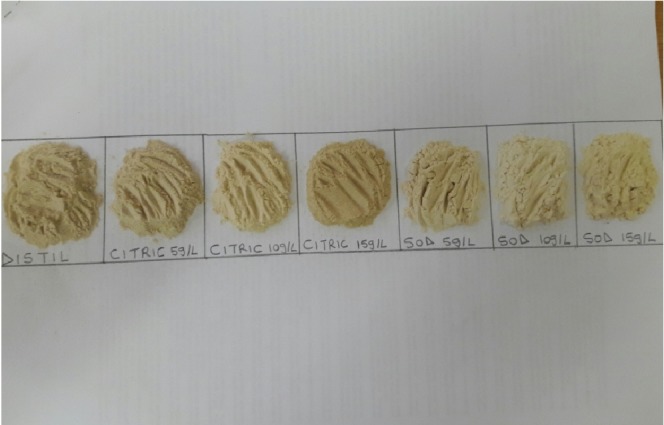
*Ndou* sweet potato flour.

*Ndou* sweet potato tubers were weighed, washed, peeled, and cut into 4 mm size. Sliced sweet potatoes were divided into two batches, and randomly assigned to two treatments (chemical agents) as follows: sodium metabisulphite and citric acid at 5, 10, and 15 g/L concentration for 10 min. The untreated (control) slices were immersed in distilled water for 10 min at room temperature. The sweet potato slices immersed in the two chemical solutions were allowed to drain for 2 min after which the pretreated and control NSP slices were put on a tray and oven dried for 10 h at 70°C. The pretreated and control NSP slices were milled into flour (Figure 1) after drying. Approximately an 80 mesh sieve was used to screen the NSP flour samples; they were stored in polyethylene bags and then kept in the refrigerator at 4°C prior to use.

### 2.4. Assessment of the Physicochemical and Functional Properties of NSP Flour

#### 2.4.1. Proximate Analysis

All proximate analyses were done in triplicate. The moisture, ash, protein and fat contents and crude fibre of NSP flour were determined using standard methods of Association of Official Analytical Chemist (AOAC) [12]. The moisture content of NSP flour was determined according to the AOAC method 945.32 with oven drying at 105°C for 3 h. Ash content was determined using the muffle furnace according to the official method 923.03. The crude protein was determined using the Kjeldahl method, AOAC method 978.02, and 6.25 × N was used to multiply the nitrogen content in order to obtain protein percentage. The fat content was determined according to the AOAC method 920.39.

#### 2.4.2. Colour Analysis

The colour of NSP flour samples was measured using a Hunterlab LabScan XE Spectrophotometer CIELAB. The black and white plates were used to calibrate the spectrophotometer and zero calibration followed. The NSP flour was put in a glass cell and placed above the light source of the instrument and colour coordinate values *L*^∗^ (Lightness/darkness), *a*^∗^ (redness/green) and *b*^∗^ (yellowness/blue) were recorded [13].

#### 2.4.3. Determination of pH

About 10 g of each sample of NSP flour samples was mixed with 100 mL of distilled water in order to measure the pH values of flour. The mixture was left at room temperature for 30 min. The pH meter that had already been standardised with buffer solutions of pH 4.0 and 7.0 was then used to measure the pH values of the supernatant.

#### 2.4.4. Water Absorption Capacity

The method of Onwuka [14] was followed to measure the water absorption capacity of NSP flour. Approximately 1 g of flour sample was weighed in a conical graduated centrifuge tube and dispersed in 10 ml of water. The mixture was thoroughly shaken for 1 min at room temperature. The sample was allowed to stand for 30 min before it was centrifuged at 5,000 × g for 30 min. The volume of free water was determined directly from the centrifuge tube. The amount of the absorbed water was multiplied by the density of water (1 g/ml) and results were expressed as g/100 g.

#### 2.4.5. Bulk Density

The method of Konak et al. [15] was followed to determine the bulk density of the flour. Approximately 50 g of NSP flour was weighed into a 100 ml graduated measuring cylinder and the cylinder was gently tapped continuously until the contents were tightly packed. The bulk density was calculated as weight of NSP flour (g) divided by NSP flour volume (cm^3^).

#### 2.4.6. Least Gelation Concentration

The method of Sathe et al. [16] was adopted to determine the least gelation concentration (LGC) of NSP flour. Sample suspensions of 2–20% (w/w) of each NSP flour samples were prepared in 10 ml distilled water and the suspension was transferred into a test tube. The test tube was heated for 1 h in boiling water and a bath of cold water was used to rapidly cool it. All the test tubes were further cooled in water at 4°C for 2 h. Inversion was done for each tube one after the other. The LGC was the concentration when the sample did not fall down or slip when inversion was done on the test tube.

#### 2.4.7. Viscosity

Approximately 10 gram of the NSP flour sample was mixed with 90 ml water at 30°C (10% slurry, w/v) and allowed to hydrate for 30 min with occasional stirring. Brookfield viscometer (Model RV, Brookfield Engineering Inc., Stoughton, USA) was used to measure the viscosity of the samples.

#### 2.4.8. Swelling Capacity and Starch Solubility Index

The method of Anyasi et al. [17] was adopted to determine the swelling capacity and solubility index of the NSP flour samples whereby about 1 g of flour was mixed with 10 mL of distilled water in a centrifuge tube and heated at 80°C for 30 min with constant stirring. The tubes were removed and cooled to room temperature. Upon cooling, samples were centrifuged for 15 min at 2200 rpm. The supernatant was decanted and weight of the puree was then determined. For solubility index, the supernatant obtained from the swelling power was decanted in a preweighed evaporation dish and dried to constant weight in the oven and calculated.

#### 2.4.9. Thermal Properties

The thermal properties of NSP flour samples were investigated as described by Sun et al. [18] with some modifications using a differential scanning calorimeter (DSC, DSC 4000, Perkin-Elmer, Shelton, CT, USA). Approximately 1 mg of NSP flour was mixed with 1 ml of distilled water placed into aluminium hermetic pans and samples were allowed to equilibrate for 1 h prior to analysis. An empty pan was used as reference. Calorimeter scan conditions were set as follows: samples were heated from 25 to 120°C with a heating rate of 10°C /min. The parameters evaluated were: Onset temperature (*T*_0_), peak time (*Tp*), conclusion temperature (*T*_*c*_) and enthalpy (Δ*H*). Pyris software (Perkin–-Elmer, Shelton, CT, USA) was used for calculation of obtained data.

### 2.5. Data Analysis

All measurements were done in triplicate. The data collected were subjected to statistical analysis to test for variance (ANOVA) using SPSS version 23.0 with Duncan test at *p* < 0.05 level of significance.

## 3. Results and Discussion

### 3.1. Physicochemical Properties of NSP Flour

The moisture content of untreated (control) NSP flour was 6.50% and it ranged from 5.54% to 7.70% for variously treated samples as shown in Table 1. This range is similar to one reported by Haile et al. [19] for an orange fleshed sweet potato cultivar. There was a significant difference of *p* < 0.05 in the moisture content of citric acid treated samples at different levels. A higher level of 7.70% of moisture content was obtained from citric acid pretreatment of 10 g/L concentration, while the lowest moisture content of 5.54% was obtained for sodium metabisulphite pretreatment of 15 g/L concentration. This might be due to the ability of sodium metabisulphite to cause dehydration in sweet potato slices. The lower moisture content of the NSP flour samples will give a better shelf life and denser nutrient [20].

All the sweet potato flour samples had the moisture content within a favourable range for effective storage of the flour with minimal risk of microbial contamination. The moisture content of flours below 14% is capable of resisting microbial growth resulting in shelf stable flour [21]. The physicochemical component is one of the principal factors that dictate flour quality, especially moisture [22, 23]. At *p* < 0.05, ash content was significantly affected by sodium metabisulphite treatment and it increased as the concentration of sodium metabisulphite increased from 1.77 to 2.54%. The increase in ash content might be due to diffusion of solutes from sodium metabisulphite solution to sweet potato slices during soaking. Ahmed et al. [8] observed similar results of ash content increase in pretreated sweet potato flour. However, citric acid did not significantly (*p* > 0.05) affect the ash content of untreated (control) and treated NSP flour samples. The low values of ash content on 15% sodium metabisulphite treated sample might be due to the leaching out of minerals during the pretreatment of NSP slices. Jangchud et al. [24] and Osundahunsi et al. [25] revealed that ash and protein contents of sweet potato flour are reduced by pretreatments involving leaching out such as blanching and parboiling. Both treatments did not significantly (*p* < 0.05) affect the protein content of NSP flour. Untreated sample (control) had the value of 2.64% while treated samples ranged from 2.54% to 2.82%. This was lower than 5% which has been reported as the crude protein content of sweet potato flour [26, 27]. However, the values are within the range of 1.0–8.5% as reported by Van Hal [1]. The decrease might be due to leaching out of nitrogenous substances during treatment. The results show that NSP flour can be used as a source of protein in rural communities to complement human diets since animal protein may be scarce or too expensive [28]. NSP flour had very little fat content. The untreated sample (control) had fat content of 0.69%. This was similar to 0.65% which was reported by Eleazu and Ironua [11]. There was no significant (*p* < 0.05) difference in sodium metabisulphite treated samples. Ahmed et al. [8] also reported sodium metabisulphite to have no significant effects on the fat content of sweet potato flour. However, citric acid significantly (*p* < 0.05) affected the fat content of NSP flour and it ranged from highest (0.68%) to lowest (0.61%). This is similar to Haile et al. [19] observations who reported that the decrease in fat of sweet potato flour samples might be due to oxidation of fat triggered by citric acid.

**Figure 2:**
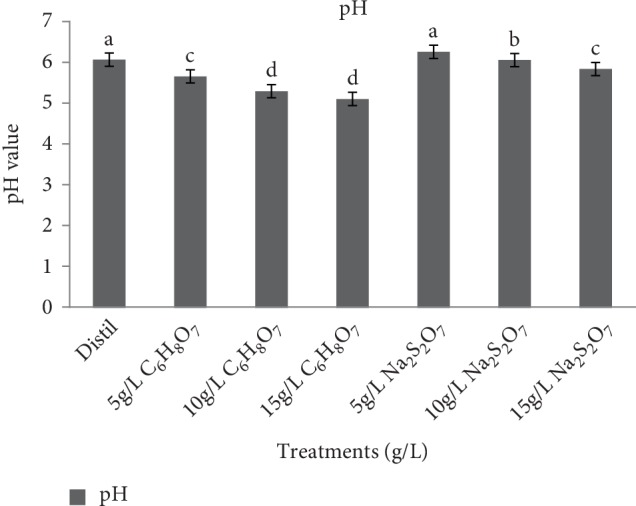
Effect of pretreatment on pH value of *Ndou* sweet potato flour. Distil = distilled water, C_6_H_8_O_7 _ = citric acid and Na_2_S_2_O_7_ = sodium metabisulphite.

The pH of a NSP flour suspension (Figure 2) is important because it affects most of the functional properties of the flour [11].There was a significant difference (*p* < 0.05) in pH values of NSP flour. The values ranged from 5.09 to 6.42. The untreated sample had a pH level of 6.05. A decrease in pH level was observed across the pretreatment concentration of sodium metabisulphite and citric acid which decreased from 6.42 to 5.82 and 5.64 to 5.09 respectively as concentration increased. Low pH values have been reported to be caused by high amylase activity which increases the level of acidity and this was similar to observations of Nabubuya et al. [29] who found the pH of creamed fleshed sweet potato flour to be 6.64. This was also in line with the observation of Haile et al.[19]. Citric acid had a greater effect on pH level of NSP flour compared to sodium metabisulphite because of its high acidity.

The results of colour measurement, as presented in Table 2 were significantly (*p* < 0.05) different in lightness (*L*^∗^), redness (*a*^∗^) and yellowness (*b*^∗^) values for all pretreated flour samples. Sodium metabisulphite increased the lightness of the flour with an increase in concentration. The increase in the *L*^∗^ value might be due to the potential of the pretreatments to retard both enzymatic and nonenzymatic reactions in the sweet potato flour. Pretreatment such as sulphite and its salts are used to preserve the colour of fruits and vegetables because of their ability to retard the reactions of both enzymatic and nonenzymatic [30]. The increase in Lightness (*L*^∗^ values) might also be due to oven drying temperature of the NSP since higher temperature inactivates phenolase enzyme. Phenolase activities have been reported to be deactivated at high temperatures [31]. Moreover, the reaction between sulphite ions and quinines inhibits phenolase activities in the flour and consumption of oxygen [1]. Ahmed et al. [8] also reported that sodium metabisulphite caused an increase in *L*^∗^ values of sweet potato flour. Compared to sodium metabisulphite, the *L*^∗^ values of citric acid treated samples were least ranging between 69.27 and 78.82 across all pretreatment concentration. Changes in carotenoids, oxidation, caramelisation, or phenolase [32] might have contributed to the decrease in *L*^∗^ values of the flour.

Conversely, the *b*^∗^ values for pretreated *Ndou* sweet potato flour was higher than the control sample in all pretreated concentration. However citric acid had a greater effect on this chroma than sodium metabisulphite. In citric acid and sodium metabisulphite, *b*^∗^ values increased as concentration level increased, ranging from 20.88 to 22.41 and 20.04 to 21.73, respectively. High yellowness values in pretreated samples can be due to the higher amount of beta carotene in NSP flour [8, 30]. Beta-carotene is a very sensitive nutrient which degrades during processing or storage and this occurs via oxidation or isomeration [1]. NSP flour could add natural colour to food products. Results obtained for the measurement of redness represented by *a*^∗^ values indicated that redness (*a*^∗^) was significantly (*p* < 0.05) affected by treatments. Sodium metabisulphite showed a decrease in redness values which ranged from 1.59 to −0.16 as concentration increased. A higher redness value of 3.74 was obtained at 10 g/L of citric acid, showing a greater intensity of redness of the flour. The higher redness value could be due to the presence of the anthocyanins pigments in the NSP flour since *β*-carotene is 6708 *μ*g/100 g in fresh sweet potatoes while anthocyanin is 525 mg/100 g fresh weight (unpublished data) and it might also have been influenced by milling conditions and other contaminants [1, 33].

### 3.2. Functional Properties of NSP Flour

**Figure 3:**
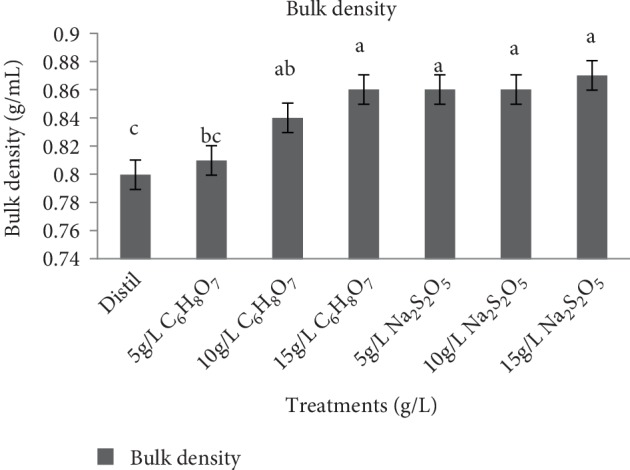
Effect of pretreatment on bulk density of *Ndou* sweet potato flour. Distil = distilled water, C_6_H_8_O_7_ = citric acid and Na_2_S_2_O_7_ = sodium metabisulphite.

The bulk density (BD) of NSP flour is shown in Figure 3. BD measures the heaviness of a flour sample. It is a property that determines the porosity of a product which influences the design of the package and is also used to determine the type of packaging material and handling, especially production of flour in the food processing industry. The BD of the untreated NSP flour (control) was 0.80 g/ml and it was generally high for the treated samples at different pretreatment concentration, ranging from 0.81 g/ml to 0.87 g/ml. There was a significant difference (*p* < 0.05) in BD for all citric acid treated samples across all pretreatment concentrations while sodium metabisulphite did not have any significant difference (*p* < 0.05) with values ranging from 0.86 to 0.87 g/ml. This was similar to the value of 0.92 g/ml obtained by Eleazu and Ironua in 2012 [11] for a cream fleshed sweet potato variety which is commercially sold in South Eastern Nigeria. The high BD of the NSP flour (0.87 g/ml) as shown in Figure 3 indicates its heaviness. This means that it might be useful in food preparations to reduce paste thickness in food products, as well as in pharmaceuticals industry as a drug binder and disintegrant [34, 35].

The results of BD revealed that it depends on the particle size and initial moisture content of flours [36]. The increase observed in this study in BD is not desirable in packaging because higher density often results in reduced ability to compress the flour. This is unlikely to result in cost saving since more packaging materials would be required.

**Figure 4:**
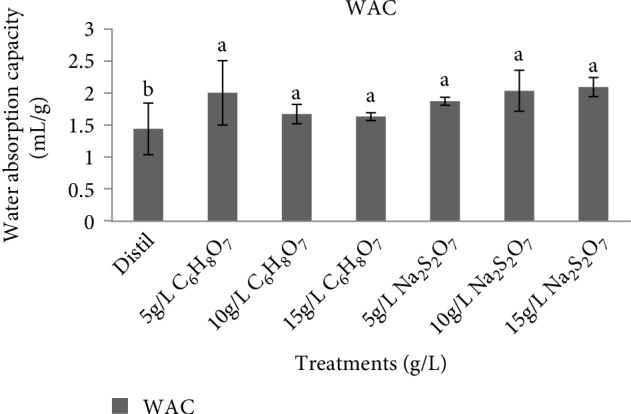
Effect of pretreatment on water absorption capacity of *Ndou* sweet potato flour. Distil= distilled water, C_6_H_8_O_7_ = citric acid and Na_2_S_2_O_7_ = sodium metabisulphite.

The water absorption capacity (WAC) of NSP flour is shown in Figure 4. Water absorption capacity is useful in determining the capacity of flour to take up water and swelling to improve uniformity in food. It is also advantageous in food processing for improving yield, uniformity, and giving shape to food products [25]. The WAC of pretreated sweet potato flour samples was significantly (*p* < 0.05) higher than that of control samples (1.44 ml/g) which ranged from 1.63 to 2.03 ml/g. The higher values of WAC recorded for the flour samples might have been caused by high polar amino acid residue of protein having affinity for water molecule [37]. Since they have hydrophilic parts such as polar or charged side chains, proteins and carbohydrates are the major chemical constituents that increase WAC of flours. The WAC of the NSP flours could also be influenced by increase in the solubility, leaching out of amylose, and loss of molecular structure of the starch as well as the crystalline structure [38].

The WAC might also be influenced by nonidentical structure of flours. The high WAC of sweet potato flours shows that they can be useful in the formulation of various foods such as meat sausage, bakery products, dough, and processed cheese [36].

**Figure 5:**
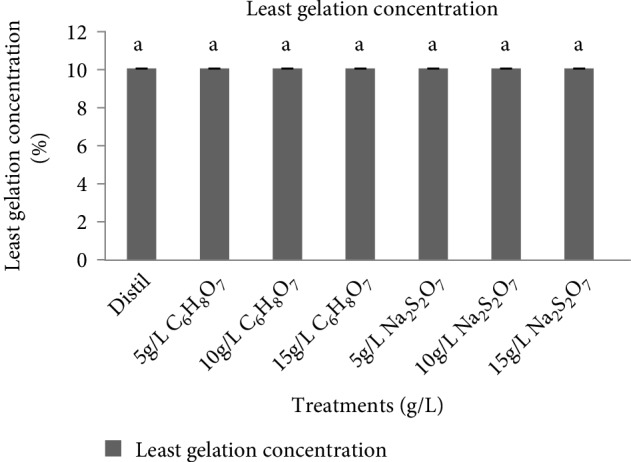
Effect of pretreatment on least gelation concentrantion of *Ndou* sweet potato flour. Distil = distilled water, C_6_H_8_O_7_ = citric acid and Na_2_S_2_O_7_ = sodium metabisulphite.

Both pretreatments did not significantly (*p* > 0.05) affect the LGC of NSP flour as shown in Figure 5. This was similar to results obtained by Chandra et al. [36] on composite wheat and sweet potato flours (8–10%, w/v) and Akubor [39] on pretreated yam flours (8%, w/v). However, all NSP flours are required to form a gel at low concentration (10%, w/v). The LGC for other flours such as safflower and maize [40] and plantain [39] were 8, 6, and 6 (w/v). The low LGC of some of these flours may be added as composite food for the curd formation or be used as additives to other food materials forming gel in food products. Better gelling ability of the protein ingredient requires lower LGC of the flourand this also results in increased swelling ability of the flour [41].

Akubor [39] reported that gelation capacity of flours is caused by protein concentration, particularly fraction of globulin, and interaction between proteins, carbohydrates, and lipids. Vautsinas and Nakai [42] reported that hydrophobicity and square of sulfhydryls of proteins significantly affect the protein gelation of the flour. The present study's LGC result shows that NSP flour can be useful as a thickening and gelling agent in food products such as sauce and puddings.

Viscosity, swelling power, and starch solubility index of NSP flour are shown in Table 3. The viscosity of NSP flour samples ranged from 26.2 to 37.1 cP with control and sodium metabisulphite pretreated sample at 10% having the lowest and highest values, respectively. Low molecular weight carbohydrates in the control sample might have contributed to reduced viscosity since they possess less water binding ability and may be more easily digested and absorbed as required by infants. Reduced viscosity in the control sample will be a good indicator for the appropriateness of a weaning food blend for infants [43]. The swelling power (SP) of pretreated NSP samples was significantly (*p* < 0.05) higher than that of control samples (7.18 g/ml) which ranged from 8.47 to 7.57 g/ml except for citric acid pretreated sample at 15% which had a lower value of 6.30 g/ml. Structural changes within the starch granules might be responsible for the increase in SC of pretreated samples [44]. When starch is gelatinised at a certain temperature, the molecular organisation is disrupted within the granules and the interactions between starch and water increase, resulting in a notable increase in the SP [45]. The presence of a large number of crystallites might have contributed to the low SP of citric acid pretreated sample at 15% since large crystallites increases granular stability thereby reducing the extent of granular swelling [46].

The decrease in SP could also be due to the interactions that might have occurred between amylose–amylose and amylo-pectin–amylopectin chains [44]. A low SP makes it more difficult for starch to be gelatinised since more mechanical energy is required to disrupt the crystallised areas of starch. The solubility index (SI) of pretreated NSP flours was significantly (*p* < 0.05) higher than that of control samples (7.20 g/ml), ranging from 7.3 to 9.6 g/ml. The difference in the SI is related to the difference in the extent of starch degradation in NSP flour samples. High SI in pretreated samples could be due to high amount of amylose which leaches out easily during the swelling process [47]. At a higher SI such as in the sample pretreated with citric acid at 10%, there is higher degradation of starch leading to more number of soluble molecules in the flour. Various factors such as source, SP, inter-associative forces within the amorphous, crystalline domains as well as presence of other components may influence solubility of starches in the flour [48].

Thermal properties of NSP flour are shown in Table 4. The onset temperature ((*T*_0_), peak temperature (*T*_*P*_), conclusion temperature (*T*_*c*_), and gelatinization Δ*H* values of NSP flour samples are given in Table 3. The onset temperature (*T*_*o*_) ranges from 51.26 to 63.65°C, peak temperature (*T*_*p*_) from 67.58 to 75.72°C, conclusion temperature (*T*_*c*_) from 74.73 to 85.28°C and enthalpy of gelatinization (Δ*H*) from 8.48 to 12.78 J/g. A significant difference (*p* < 0.05) was observed in *T*_*o*_, *T*_*p*_, and *T*_*c*_ and of pretreated and control NSP flour samples.

Low transition temperatures and Δ*H* values recorded in NSP flour samples might be attributed to large quantities of short amylopectin chains present in the starch of the flours [49, 50] while slightly higher transition temperature values of the samples might be due to the contribution of heat-moisture treatment rather than pretreatments used in this study [51]. Shinoj et al. [52] reported that high transition temperatures are expected to result from a high degree of crystallinity, which impacts structural stability by making the starch granules more resistant to gelatinisation. This means that chemically pretreated NSP flour samples were more structurally stable and more resistant to gelatinisation. Both citric acid and sodium metabisulphite interacted with the matrix of NSP flour which caused a release of water and matric hardening resulting in a higher gelatinisation temperature.

### 3.3. Correlation of NSP Flour Samples

The correlation between functional and physicochemical properties of NSP flour is represented in Table 5. Lightness was positively correlated to WAC, BD pH and negatively correlated to LGC. This suggested that the higher the lightness of the flour, the higher WAC, BD, pH and the lower LGC it will have. The least gelation concentration was 99% which negatively correlated to pH level of NSP flour. Redness was observed to be negatively correlated to water absorption capacity, pH level, bulk density and lightness which means that as these parameters increase, redness decreases and vice versa. Yellowness of the flour was found to increase with an increase in WAC, pH, redness and least gelation concentration and decrease with an increase in lightness and bulk density.

Correlation among proximate composition of NSP flour is shown in Table 6. The results show that ash content is inversely correlated to moisture content and also that there is 99% possibility that fat content increases with an increase in moisture content. Protein was negatively correlated to fat, ash and moisture content such that when those parameters increase, protein content decreases and vice versa. Ash and fat contents have also been found to be negatively correlated.

Correlation between proximate composition, physicochemical, and functional properties of NSP flour is shown in Table 7. The results show that fat content of the flour is negatively correlated to water absorption capacity, pH level, bulk density, and lightness. This means that flour with high fat content cannot hold high water as compared to the flour with low fat content. The results also show that protein content increases with a decrease in water absorption capacity, pH level, bulk density, redness, and yellowness of the flour. Ash content was 99% positively correlated to redness and negatively correlated yellowness of NSP flour.

## 4. Conclusions

NSP flour was prepared by pretreating the tubers with citric acid and sodium metabisulphite at three different concentrations of 5, 10, and 15 g/L for 10 min. In the conversion of dried pretreated NSP slices to flour, citric acid had a significant effect on moisture, bulk density, pH, and colour while sodium metabisulphite had a significant effect on moisture, ash, pH, and colour. Both citric acid and sodium metabisulphite did not have any significant effects on protein, fat, water absorption capacity, and least gelation concentration. Pretreatments did improve physicochemical and functional properties of NSP flour. However, sodium metabisulphite was more effective than citric acid at 15 g/L. It is, therefore, recommended that a further study should be conducted on the effect of pretreatments on some bioactive compounds and other functional properties such as oil absorption capacity, emulsion activity, and foam capacity and stability of NSP flour.

## Figures and Tables

**Table 1:** Proximate composition of NSP flour.

Proxi (%)	Treatments (g/L)						
	Distil		C_6_H_8_O_7_			Na_2_S_2_O_7_	
		5	10	15	5	10	15
Moisture	6.50 ± 0.71^bc^	5.98 ± 0.14^c^	7.15 ± 0.56^ab^	7.70 ± 0.13^a^	6.61 ± 0.02^b^	6.85 ± 0.17^b^	5.54 ± 0.71^d^
Ash	1.03 ± 0.03^c^	1.04 ± 0.02^c^	1.08 ± 0.07^c^	1.07 ± 0.07^c^	1.77 ± 0.13^b^	2.54 ± 0.13^a^	0.70 ± 0.05^d^
Protein	2.64 ± 0.15^ab^	2.54 ± 0.02^b^	2.54 ± 0.04^b^	2.72 ± 0.08^a^	2.07 ± 0.07^c^	2.62 ± 0.16^b^	0.68 ± 0.03^d^
Fat	0.69 ± 0.02^b^	0.68 ± 0.01^b^	0.65 ± 0.01^b^	0.61 ± 0.02^b^	2.87 ± 0.04^a^	2.82 ± 0.17^a^	0.68 ± 0.03^b^

Results are mean ± standard deviation. Values in the same row followed by different letters are significantly different (*p* < 0.05). Distil: distill water, C_6_H_8_O_7_ = citric acid, Na_2_S_2_O_7_ = sodium metabisulphite and Proxi = proximate composition.

**Table 2:** Colour attributes of *N*SP flour samples.

Color attributes	Treatment (g/L)						
	Distil	C_6_H_8_O_7_			Na_2_S_2_O_7_		
		5	10	15	5	10	15
*L* ^∗^	80.71 ± 0.05^d^	69.27 ± 1.11^ g^	72.82 ± 0.24^f^	78.82 ± 0.27^e^	83.58 ± 0.21^c^	85.51 ± 0.16^b^	86.79 ± 0.11^a^
*a* ^∗^	1.27 ± 0.03^d^	2.98 ± 0.10^b^	3.74 ± 0.31^a^	1.59 ± 0.08^c^	0.80 ± 0.07^d^	0.24 ± 0.05^e^	-0.16 ± 0.04^f^
*b* ^∗^	18.26 ± 0.05^c^	20.88 ± 0.49^b^	22.24 ± 1.09^a^	22.41 ± 0.09^a^	20.04 ± 0.26^b^	20.58 ± 0.19^b^	21.73 ± 0.16^a^

Results are means ± standard deviation (*n* = 3). Values across rows followed by different letters are significantly different (*p* < 0.05). Distil: distill water C_6_H_8_O_7_: citric acid and Na_2_S_2_O_7_: Sodium metabisulphite, *L*^∗^: lightness, *a*^∗^: redness (+60) & (−60) greenness and *b*^∗^: yellowness (+60) & (−60) blueness.

**Table 3:** Viscosity, swelling power and solubility index of NSP flour.

Functional properties	Treatments (g/L)						
	Distil	C_6_H_8_O_7_			Na_2_S_2_O_7_		
		5	10	15	5	10	15
Viscosity (cP)	26.2 ± 0.61^d^	33.3 ± 0.62^b^	36.4 ± 0.62^a^	29.1 ± 0.60^c^	28.3 ± 0.61^c^	37.1 ± 0.62^a^	36.0 ± 0.09^a^
SP (g/ml)	7.18 ± 1.61^e^	8.47 ± 1.82^b^	9.59 ± 0.51^a^	6.30 ± 0.41^d^	8.05 ± 1.21^c^	7.77 ± 0.22^d^	7.57 ± 0.91^d^
SI (g/ml)	7.20 ± 1.61^e^	8.4 ± 1.81^b^	9.6 ± 0.52^a^	7.30 ± 0.41^d^	8.0 ± 1.22^b^	7.82 ± 0.21^c^	7.61 ± 0.91^d^

Results are mean ± standard deviation. Values in the same row followed by different letters are significantly different (*p* < 0.05). Distil: distill water, C_6_H_8_O_7_ = citric acid, Na_2_S_2_O_7_ = sodium metabisulphite, SP = Swelling power and SI = solubility index.

**Table 4:** Thermal properties of NSP flour.

Thermal properties (g/L)	Treatments						
	Distil	C_6_H_8_O_7_			Na_2_S_2_O_7_		
		5	10	15	5	10	15
*T*_*O*_ (°C)	51.26 ± 2.15^d^	56.28 ± 0.67^c^	60.92 ± 1.71^b^	57.81 ± 1.91^c^	63.40 ± 1.06^a^	58.12 ± 2.02^c^	63.65 ± 6.35^a^
*T*_*p*_ (°C)	67.58 ± 1.97^d^	70.83 ± 0.25^b^	72.65 ± 1.26^b^	69.30 ± 1.56^c^	75.72 ± 1.56^a^	70.88 ± 2.46^b^	73.26 ± 5.25^a^
*T*_*c*_ (°C)	74.73 ± 2.64^c^	84.35 ± 0.46^a^	85.38 ± 1.05^a^	77.58 ± 3.56^b^	85.28 ± 5.60^a^	83.17 ± 3.14^a^	81.38 ± 2.86^b^
Δ*H* (J/G)	8.43 ± 3.38	12.78 ± 0.23	9.89 ± 1.63	10.05 ± 2.14	9.15 ± 3.65	9.63 ± 2.94	8.76 ± 1.92

Results are mean ± standard deviation. Values in the same row followed by different letters are significantly different (*p* < 0.05). Distil: distilled water, C_6_H_8_O_7_ = citric acid, Na_2_S_2_O_7 _= sodium metabisulphite, *T*_*O*_, Onset temperature, *T*_*p*_, peak temperature, *T*_*c*_, conclusion temperature, Δ*H*, enthalpy of gelatinisation.

**Table 5:** Pearson correlation matrices between functional properties and physicochemical properties of NSP flour samples.

	WAC	pH	BD	LGC	*L* ^∗^	*a* ^∗^	*b* ^∗^
WAC							
pH	0.082						
BD	0.401	−0.190					
LGC	−0.094	−0.606^∗∗^	−0.129				
L	0.190	0.331	0.453^∗^	−0.265			
a^∗^	−0.183	−0.291	−0.458^∗^	0.215	−0.997^∗∗^		
b^∗^	0.291	0.386	−0.238	0.083	−0.238	0.249	

^∗∗^Correlation is significant at the 0.01 level (2-tailed) and ^∗^Correlation is significant at the 0.05 level (2-tailed). WAC = water absorption capacity, BD = bulk density, LGT = least gelation concentration, *L*^∗^ = lightness, *a*^∗^ = redness and *b*^∗^ = yellowness.

**Table 6:** Pearson correlation matrices of proximate composition of NSP flour samples.

	Fat	Ash	*MC*	*PC*
Fat				
Ash	−0.408			
*MC*	0.728^∗∗^	−0.075		
*PC*	−0.107	−0.170	−0.106	

^∗∗^Correlation is significant at the 0.01 level (2-tailed) and ^∗^Correlation is significant at the 0.05 level (2-tailed). MC = moisture content and PC = protein content.

**Table 7:** Pearson correlation matrices among proximate composition, physicochemical and functional properties of NSP flour samples.

	WAC	pH	BD	LGT	*L* ^∗^	*a* ^∗^	*b* ^∗^
Fat	−0.036	−0.556^∗∗^	−0.269	0.381	−0.923^∗∗^	0.905^∗∗^	0.274
Ash	−0.325	−0.054	−0.436^∗^	−0.326	−0.574^∗∗^	−0.603^∗∗^	−0.076
MC	0.322	−0.276	−0.030	0.311	−0.536^∗^	0.515^∗^	0.733^∗∗^
PC	−0.278	−0.255	−0.300	0.473^∗^	0.214	−0.254	−0.161

^∗∗^Correlation is significant at the 0.01 level (2-tailed) and ^∗^Correlation is significant at the 0.05 level (2-tailed). WAC = water absorption capacity, BD = bulk density, PC: protein content, LGT = least gelation concentration, *L*^∗^ = lightness, *a*^∗^ = redness and *b*^∗^ = yellowness.

## Data Availability

The data used to support the findings of this study are available from the corresponding author upon request.
